# The Construction and Application of a New Screening Method for Phosphodiesterase Inhibitors

**DOI:** 10.3390/bios14050252

**Published:** 2024-05-16

**Authors:** Chunhua Gao, Zhe Wang, Xiaojing Liu, Rongzhen Sun, Shengyao Ma, Zongchen Ma, Qi Wang, Guoqiang Li, Han-Ting Zhang

**Affiliations:** 1 Department of Pharmacology, School of Pharmacy, Qingdao University, Qingdao 266073, China; gaochunhua@qdu.edu.cn (C.G.); wangzhe@qdu.edu.cn (Z.W.); liuxiaojing2@qdu.edu.cn (X.L.); sunrongzhen@qdu.edu.cn (R.S.); mashengyao@qdu.edu.cn (S.M.); 2 Key Laboratory of Marine Drugs, Chinese Ministry of Education, School of Medicine and Pharmacy, Ocean University, Qingdao 266003, China; mazongchen@stu.ouc.edu.cn

**Keywords:** phosphodiesterases, cyclic adenosine monophosphate, cyclic guanosine monophosphate, inhibitors, screening methods, compounds

## Abstract

Phosphodiesterases (PDEs), a superfamily of enzymes that hydrolyze cyclic adenosine monophosphate (cAMP) and cyclic guanosine monophosphate (cGMP), are recognized as a therapeutic target for various diseases. However, the current screening methods for PDE inhibitors usually experience problems due to complex operations and/or high costs, which are not conducive to drug development in respect of this target. In this study, a new method for screening PDE inhibitors based on GloSensor technology was successfully established and applied, resulting in the discovery of several novel compounds of different structural types with PDE inhibitory activity. Compared with traditional screening methods, this method is low-cost, capable of dynamically detecting changes in substrate concentration in live cells, and can be used to preliminarily determine the type of PDEs affected by the detected active compounds, making it more suitable for high-throughput screening for PDE inhibitors.

## 1. Introduction

Phosphodiesterases (PDEs), a superfamily of enzymes capable of hydrolyzing cyclic adenosine monophosphate (cAMP) and/or cyclic guanosine monophosphate (cGMP), are involved in regulating the content of second messengers [1]. According to their structural similarity, hydrolysis characteristics, and cellular and subcellular distribution, mammalian PDEs can be divided into 11 families (PDE1–11). Among them, PDE4, 7, and 8 primarily hydrolyze cAMP, and PDE5, 6, and 9 specifically hydrolyze cGMP, while the others are not selective for either [2]. Due to the close correlation between PDE activity and intracellular second messenger contents, PDE inhibitors play an influential role in the treatment of numerous diseases, especially neurological disorders [3]. For example, the PDE4 inhibitor roflumilast was the first developed treatment for specific phenotypes of chronic obstructive pulmonary disease (COPD) [4], and the PDE5 inhibitor sildenafil is an orally effective therapy for the treatment of males with erectile dysfunction (ED) [5]. On account of the wide range of diseases involved, the development of inhibitors targeting PDE has become a research hotspot.

To identify increasingly effective PDE inhibitors, it is particularly important to choose appropriate screening methods. At present, the screening methods for PDE inhibitors mainly include ^3^H adenine pre-labeling [6], enzyme-linked immunosorbent assay (ELISA) [7], fluorescence resonance energy transfer (FRET) [8], bioluminescence resonance energy transfer (BRET) [9], and PDE-Glo ™ determination [10]. The ^3^H adenine pre-labeling method uses radioactive isotopes to label cAMP/cGMP, detect the number of remaining substrates, and determine enzyme activity [11]. This method has the advantages of high sensitivity and a wide dynamic range, but radioactive isotopes are harmful to human health and the environment, requiring extremely strict experimental conditions and procedures [12,13]. ELISA is based on antigen–antibody reactions and is used to detect the amount of cAMP or cGMP [14]. This method is easy to use, has high sensitivity and specificity, and does not involve radioactive substances; however, the use of antibodies makes it costly [15]. FRET describes the non-radiative transfer of energy stored in excited fluorescent molecules (donors) to nearby non-excited fluorescent molecules (acceptors) [16]. In contrast, BRET involves luciferase instead of fluorescent protein as a donor and does not require laser excitation but rather emits light after substrate molecule oxidation [17]. Compared with antibody-based methods, FRET and BRET detections have higher spatial and temporal resolutions. However, their cytotoxicity makes them unsuitable for long-term imaging, and the narrow detection limit also limits their use [18]. PDE-Glo ™ determination is a method to detect the activity of PDEs in vitro. The principle of this method is that binding to cNMP causes PKA to be activated, consuming ATP in the process. The level of remaining ATP is determined using the luciferase-based Kinase-Glo^®^ Reagent. As PDEs hydrolyze cAMP or cGMP, respectively, the concentration of cAMP or cGMP decreases, and more ATP is available for luciferase reactions. Therefore, luminescence is proportional to PDE activity [19]. PDE-Glo™ determination can directly reflect the impact of the tested sample on PDE activity, but this method requires expensive reagents and involves strict requirements with respect to reagent storage and experimental conditions [20]. This makes the method unsuitable for the high-throughput screening of PDE inhibitors.

Based on the above issues, the development of an efficient and cost-effective screening method for PDE inhibitors is crucial for the development of new drugs. GloSensor technology can be used to construct a recombinant plasmid capable of expressing luciferase from firefly *Photonus pyralis*, during which the cAMP-binding domain B from protein kinase A (PKA) regulatory subunit type IIβ (RIIβB) is inserted near the hinge region of the luciferase. As a result, the mutant luciferase expressed by the plasmid transforms into an active conformation by binding to cAMP, catalyzing the substrate luciferin’s luminescence. The magnitude of the luminescence signal reflects the level of intracellular cAMP [21]. This technology has been mainly applied to research related to G protein-coupled receptors (GPCRs) [22]. The design of cGMP sensors is based on a similar principle and involves replacing the cAMP binding domain with a cGMP binding domain [23]. Since cAMP and/or cGMP are hydrolytic substrates of PDEs, the content of intracellular cAMP and/or cGMP can be detected by GloSensor technology to reflect the activity of PDEs. In the present study, a new PDE inhibitor screening method based on GloSensor technology was established and successfully applied to drug screening, leading to the discovery of several compounds with different structural types and PDE inhibitory activity. Compared with previous methods, the new method has advantages due to the easy availability of raw materials, simple operation, and the ability to dynamically monitor changes in intracellular cAMP and cGMP levels. It can also be used to preliminarily determine the PDE types of active compounds, making it more suitable for the high-throughput screening of PDE inhibitors.

## 2. Materials and Methods

In this study, cell models were constructed for the high-throughput screening of PDE inhibitors. To further demonstrate their effectiveness, the reported PDE inhibitors were measured using this method. Subsequently, the method was applied for compound screening, and in vitro enzyme activity testing was performed on the screened compounds.

### 2.1. Materials and Solutions

Penicillin–Streptomycin solution (10,000 U/mL Penicillin, 10,000 μg/mL Streptomycin; Cat #C100C5) was obtained from NCM Biotech (Suzhou, China). Fetal Bovine Serum (FBS; Cat #BC-SE-FBS01) and phosphate-buffered saline (PBS; Cat #10010001) were obtained from Sbjbio (Nanjing, China). Trypsin-EDTA Solution (Cat #BL512A) was obtained from Biosharp (Hefei, China). DMEM medium (High Glucose; Cat #XB01-01) was obtained from VivaCell (Shanghai, China). Opti-MEM medium (Cat #Lvn1012A) was obtained from Livning (Beijing, China). Forskolin (Coleonol; Cat #66575-29-9), S-Nitroso-N-acetyl-DL-penicillamine (SNAP; Cat #67776-06-1), Isobutylmethylxanthine (IBMX; Cat #28822-58-4), PF-05180999 (PF-999; Cat #1394033-54-5), rolipram (Cat #61413-54-5), sildenafil (UK-92480; Cat #139755-83-2), icariin (Cat #489-32-7), and D-Luciferin sodium (Cat #103404-75-7) were purchased from MedChemExpress (Shanghai, China). The Lipofectamine™ 2000 Transfection Kit (Cat #11668500) was obtained from Thermo Fisher Scientific (Waltham, MA, USA). The pGloSensor™-22F cAMP Plasmid map (Cat #E2301), pGloSensor™-42F cGMP Plasmid map (Cat #CS177001), and PDE-Glo™ Phosphodiesterase Assay (Cat #V1361) were purchased from Promega (Madison, WI, USA). Recombinant human PDE4B protein (Cat #ab125582) and recombinant human PDE7A/HCP1 protein (Cat #ab125786) were obtained from Abcam (Cambridgeshire, UK). Compounds **1**–**7** were provided by Guoqiang Li’s research group (School of Medicine and Pharmacy, Ocean University, Qingdao, China).

### 2.2. Cell Culture

The HEK293T human embryonic kidney cell line was purchased from SunnCell (Wuhan, China). After recovery, HEK293T cells were passaged 3–4 times a week and cultured at 37 °C in 5% CO_2_. The culture medium consisted of DMEM, 10% FBS, and 1% antibiotic solution.

### 2.3. Cell Transfection

Cells were grown to a density of 70–80% in a 6 cm culture dish, the original culture medium was discarded, and 3 mL Opti-MEM was added. A total of 5 μL lipo2000 transfection reagents and 7 μL plasmids were each diluted to 50 μL with Opti-MEM. The above two solutions were mixed, allowed to stand for 20 min, and then added to the cell culture medium. They were then incubated with 5% CO_2_ at 37 °C for 5 h. The original culture medium was discarded and replaced with the DMEM culture medium. After overnight cultivation at 37 °C and 5% CO_2_, the transfected cells were transferred to a 96-well plate and cultured under the same conditions for 9 h.

### 2.4. Live Cell cAMP/cGMP Measurement

The original culture medium was replaced with 80 μL/well loaded culture medium (containing 2 mM D-Luciferin protein) and incubated at room temperature (20–23 °C) in the dark for 2 h. A total of 10 μL/well with a concentration of 50 μM forskolin (for cAMP testing) or 250 μM SNAP (for cGMP testing) was added to the sample culture medium to increase the level of background cyclic nucleotides in the cells. According to the detection requirements, a certain concentration of analytes was sequentially added to different wells, and the luminescence signal was measured using the SynergyNeo2 (Agilent, Santa Clara, CA, USA) instrument at time 0. The measurement was conducted every 5 min, and the longest duration was based on the start of the luminescence signal’s decline.

### 2.5. In Vitro Enzymatic Activity Assay

The inhibitory activities of the compounds against PDE4B and PDE7A were determined according to the procedures required for the PDE-Glo™ Phosphodiesterase Assay.

### 2.6. Data Analysis and Statistics

Data analysis was carried out using Microsoft Excel and GraphPad Prism 8 software (version 8.0.2 for Windows, GraphPad Software, San Diego, CA, USA). The data in the luminescence signal from the replica well were averaged to produce a response vs. time curve. The cell screening results of the compounds were expressed as percentages (the difference in luminescence signals between the experimental group and the blank control group/blank control group luminescence signal × 100%). For the measurement of IC_50_, at least seven concentrations of chemicals were used, and IC_50_ values were calculated using the nonlinear method.

## 3. Results

### 3.1. Construction of the PDE Inhibitor Screening Method

#### 3.1.1. The Necessity and Optimal Concentration of AC/GC Agonists

**Figure 1:**
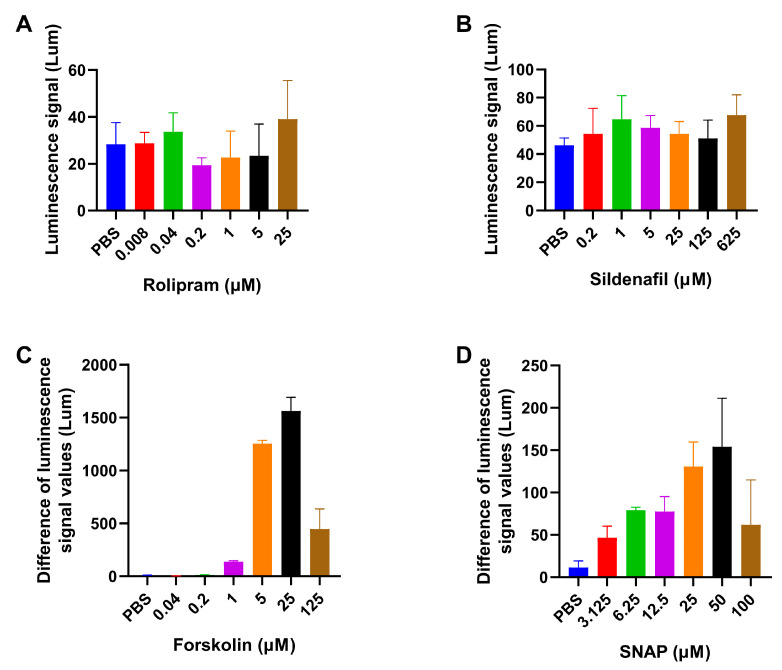
The effect of AC/GC agonists. (**A**) Detection results of the PDE4 inhibitor rolipram with gradient concentrations (blue: 0 μM; red: 0.008 μM; green: 0.04 μM; purple: 0.2 μM; orange: 1 μM; black: 5 μM; brown: 25 μM) in the cell screening model for cAMP. (**B**) The detection results of the PDE5 inhibitor sildenafil with gradient concentrations (blue: 0 μM; red: 0.2 μM; green: 1 μM; purple: 5 μM; orange: 25 μM; black: 125 μM; brown: 625 μM) in the cell screening model for cGMP. (**C**) When different concentrations of the AC agonist forskolin (blue: 0 μM; red: 0.04 μM; green: 0.2 μM; purple: 1 μM; orange: 5 μM; black: 25 μM; brown: 125 μM) were added, the cell screening model for cAMP showed the difference in luminescence signal values between rolipram (5 μM) and PBS (Z-factor = 0.8 in 5 μM forskolin). (**D**) When different concentrations of the GC agonist SNAP (blue: 0 μM; red: 3.125 μM; green: 6.25 μM; purple: 12.5 μM; orange: 25 μM; black: 50 μM; brown: 100 μM) were added, the cell screening model for cGMP showed the difference in luminescence signal values between sildenafil (10 μM) and PBS (Z-factor = 0.5 in 25 μM SNAP). The data represent the highest luminescence signals detected and are expressed as means ± standard errors (*n* = 3).

In the early stages of the experiment, a 96-well plate with transfected HEK293T cells was used directly to measure the activity of PDE inhibitors. Rolipram is a selective PDE4 inhibitor [24] and sildenafil is potent PDE5 inhibitor [25]. However, after testing, it was found that even with higher concentrations of the inhibitors, changes in cyclic nucleotide levels in cells were difficult to detect (Figure 1A,B). This might be due to the low contents of cAMP/cGMP in cells, which resulted in a relatively small change in the hydrolysis amount of cAMP/cGMP after PDE inhibitors took effect. Therefore, before adding the sample, the AC agonist forskolin or the GC agonist S-Nitroso-N-acetyl-DL-penicillamine (SNAP) was added to the cells to increase the intracellular background of cAMP/cGMP levels, and further observation was conducted to detect the cell model ability for PDE inhibitors.

To explore the optimal reaction concentration of AC agonists, cells before sample addition were treated with different final concentrations of forskolin (0, 0.04, 0.2, 1, 5, 25, 125 μM), and rolipram with a final concentration of 5 μM was added. The addition of rolipram had virtually no effect on the detected luminescence signal when there was no forskolin or when the concentration of forskolin was low. When the final concentration of forskolin was 5 μM, there was a significant difference between the rolipram and PBS groups, and the difference in their luminescence signal values reached over 1000. On this basis, the concentration of forskolin continued to increase, and the difference between the rolipram and control groups remained unchanged or even decreased (Figure 1C). Considering the detection efficiency, the possible toxicity of the reagent to cells, and the Z-factor, the optimal concentration of the AC agonist forskolin required for the cell screening model targeting cAMP was set at 5 μM.

Cells were treated with SNAP at different final concentrations (0, 3.125, 6.25, 12.5, 25, 50, 100 μM), followed by the addition of sildenafil at a final concentration of 10 μM. The results are shown in Figure 1D. Similar to the cell screening models targeting cAMP, the cell screening models targeting cGMP could not effectively detect the ability of sildenafil to increase intracellular cGMP levels when the GC agonist SNAP was not present or was at low concentrations. When the SNAP concentration was overly steep, the difference between the sildenafil and control groups narrowed, which was not conducive to the detection results. Based on the same considerations, the optimal concentration of SNAP required for the cell screening model targeting cGMP was set at 25 μM.

#### 3.1.2. Determination of the Shortest Detection Time for Cell Screening Models

**Figure 2:**
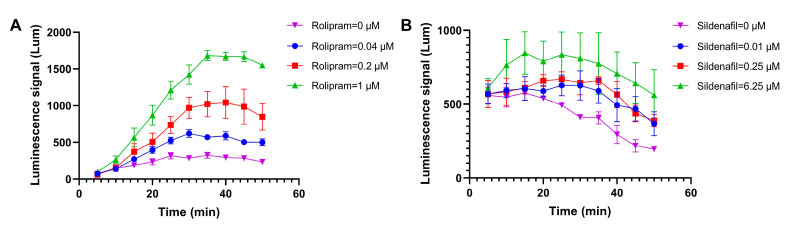
The luminescence signal–time curve in two cell models. (**A**) The cAMP cell screening model; the changes in detection values of different concentrations of rolipram over time. (**B**) The cGMP cell screening model; the detection values of different concentrations of sildenafil over time. The data are represented as means ± standard errors (*n* = 3).

To determine the shortest detection time for the two cell models, the luminescence signal–time curve was established. Based on the variations in luminescence detection values over time from two sets of data (Figure 2), it was determined that the detection time for cell screening models targeting cAMP should not be less than 35 min, while the detection time for cell screening models targeting cGMP should not be less than 30 min.

### 3.2. Validation of the Effectiveness of the PDE Inhibitor Screening Method

To verify the effectiveness of the PDE inhibitor screening method that was developed, the activity of the different types of PDE inhibitors reported earlier in the paper was measured using this method.

#### 3.2.1. The Detection Results for Positive Control Drugs

**Figure 3:**
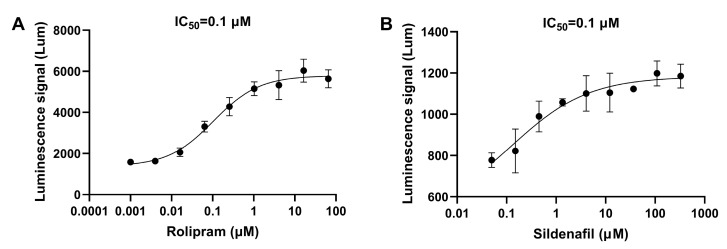
The IC_50_ curves of rolipram and sildenafil. (**A**) The IC_50_ curve of the synthetic PDE4 inhibitor rolipram detected using the constructed cell screening model targeting cAMP. (**B**) The IC_50_ curve of the synthetic PDE5 inhibitor sildenafil detected using the constructed cell screening model targeting cGMP. The data are expressed as means ± standard errors (*n* = 3).

**Figure 4:**
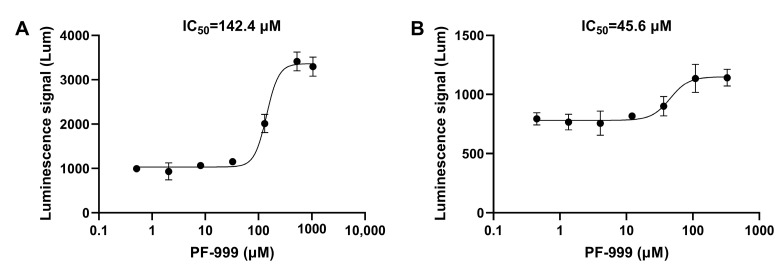
The IC_50_ curves of PF-999. (**A**) The IC_50_ curve of the PDE2 inhibitor PF-999 detected using the constructed cell screening model targeting cAMP. (**B**) The IC_50_ curve of PF-999 detected using the constructed cell screening model targeting cGMP. The data are expressed as means ± standard errors (*n* = 3).

**Figure 5:**
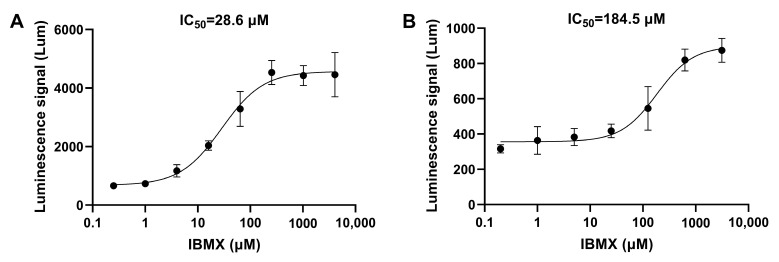
The IC_50_ curves of IBMX. (**A**) The IC_50_ curve of the non-selective PDE inhibitor IBMX detected using the constructed cell screening model targeting cAMP. (**B**) The IC_50_ curve of IBMX detected using the constructed cell screening model targeting cGMP. The data are expressed as means ± standard errors (*n* = 3).

Firstly, the cAMP-specific PDE inhibitor rolipram and the cGMP-specific PDE inhibitor sildenafil were used for validation. The IC_50_ of both inhibitors detected using this method was around 0.1 μM (Figure 3). The method was also tested for some other types of PDE inhibitors. PDE2 is a dual-substrate PDE that hydrolyzes both cAMP and cGMP [26], and PF-05180999 (PF-999) is an inhibitor of PDE2A [27]. The activity of this inhibitor was tested using two cell models, and the experimental results are shown in Figure 4. Compared to cAMP-related PDEs, PF-999 had stronger inhibitory activity on cGMP-related PDEs. Isobutylmethylxanthine (IBMX) is a broad-spectrum PDE inhibitor that inhibits PDE3, PDE4, and PDE5 [28]. The IC_50_ detection results for this inhibitor in two cell models were 28.6 μM and 184.5 μM (Figure 5).

#### 3.2.2. The Detection Results for Reported Natural Products with PDE Inhibitory Activity

**Figure 6:**
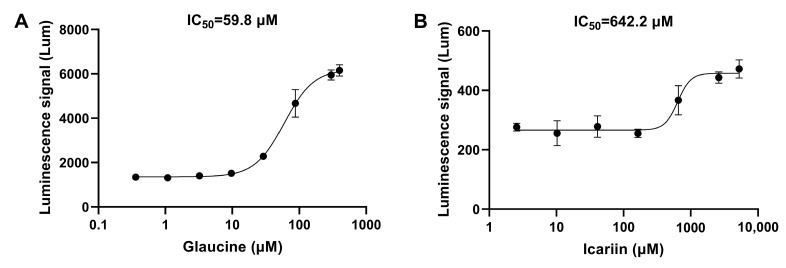
The IC_50_ curves of glaucine and icariin. (**A**) The IC_50_ curve of the natural PDE4 inhibitor glaucine detected via the constructed cell screening model targeting cAMP. (**B**) The IC_50_ curve of the natural PDE5 inhibitor icariin detected via the constructed cell screening model targeting cGMP. The data are expressed as means ± standard errors (*n* = 3).

To verify the effective application of the method in the actual screening process, a batch of reported natural products with potent PDE inhibitory activity was also detected. Glaucine (O, O-Dimethyllipoldine) is an alkaloid isolated from *Glaucium flavum* and a selective PDE4 inhibitor [29]. Icariin is a flavonol glycoside with an IC_50_ of 432 nM that inhibits PDE5 [30]. These two natural products were tested for PDE inhibitory activity using two different cell models (Figure 6). Although the detected IC_50_ was higher than the reported value, the model still predicted the inhibitory activity of the compounds.

### 3.3. The PDE Inhibitory Activity of Compounds Obtained Using the New Method

**Figure 7:**
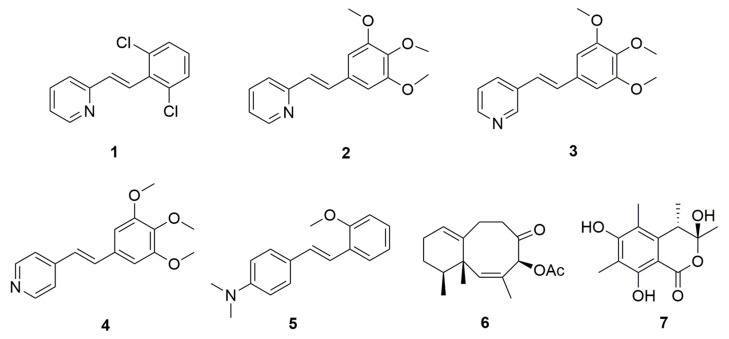
Chemical structures of active compounds selected using the new method. Compound **1**: 2-[2-(2,6-Dichlorophenyl)-ethenyl] pyridine; Compound **2**: 2-[2-(3,4,5-Trimethoxyphenyl)-ethenyl] pyridine; Compound **3**: 3-[2-(3,4,5-Trimethoxyphenyl)-ethenyl] pyridine; Compound **4**: 4-[2-(3,4,5-Trimethoxyphenyl)-ethenyl] pyridine; Compound **5**: 4-[2-(2-Methoxyphenyl)-ethenyl]-N,N-dimethylbenzenamine; Compound **6**: 4-acetoxy-2,8-neolemnadien-5-one; Compound **7**: (3*S*,4*S*)-sclerotinin A.

After the effectiveness of the new screening method was verified, it was immediately used to screen the PDE inhibitory activity of a batch of compounds with different structural types. The results showed that some compounds exhibited promising efficacy in cellular screening models targeting cAMP (Figure 7; Table 1). Through in vitro enzyme activity experiments, these compounds showed varying degrees of inhibitory activity against PDE4B and PDE7A (Table 2). Among several synthetic sources of stilbene compounds, compound **3** had fairly strong inhibitory activity on both PDE4B and PDE7A, with IC_50_ values of 5.7 μM and 4.5 μM, respectively. In addition, compounds **4** and **5** effectively inhibited PDE7A, with IC_50_ values of 8.6 μM and 2.1 μM, respectively. Further, a sesquiterpene (hexaoctagonal structure) derived from soft coral had weak PDE7A inhibitory activity, while a coumarin compound derived from microorganisms had weak PDE4B inhibitory activity but some PDE7A inhibitory activity, with an IC_50_ of approximately 5.6 μM.

## 4. Discussion

**Figure 8:**
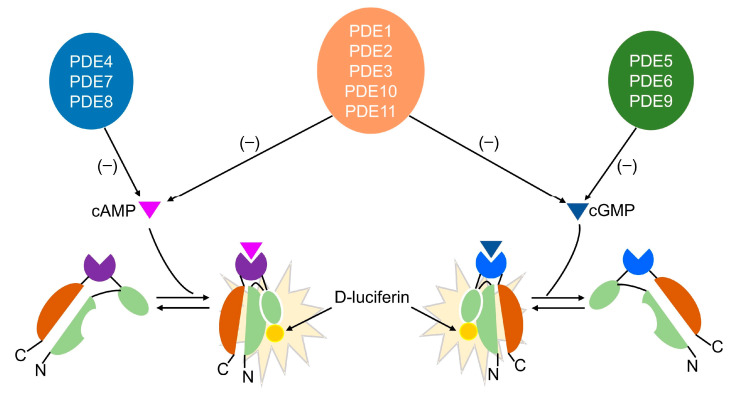
Principles of the new method. GloSensor technology constructed two recombinant plasmids capable of expressing luciferase from firefly *Photonus pyralis*, during which the cAMP or cGMP binding domain from the human type II-beta regulatory subunit of PKA (RIIβB) was inserted near the hinge region of the luciferase. As a result, the mutant luciferase expressed by the plasmid transformed into an active conformation by binding to cAMP (purple triangle) or cGMP (blue triangle), catalyzing the substrate luciferin’s luminescence (yellow circle). The magnitude of the luminescence signal reflects the level of cAMP or cGMP. If the added compound inhibits the hydrolysis of cAMP or cGMP by PDE, the detected luminescence signal will increase.

In this study, a new method screening for PDE inhibitors was developed based on GloSensor technology. This technology was originally developed by Promega; two plasmids were designed to encode a biosensor variant with a cAMP or cGMP binding domain fused to a mutant form of Photinus pyralis luciferase. Upon binding to cAMP or cGMP, the enzyme transforms into an active conformation, catalyzing D-luciferin, resulting in a significant increase in light output. The two reporter cell lines were constructed by transfecting pGloSensor™-22F cAMP plasmid or pGloSensor™-42F cGMP plasmid into HEK293T cells. Given the hydrolysis effect of PDE on cAMP or cGMP, transfected cells can detect changes in intracellular cAMP or cGMP levels after the addition of PDE inhibitors, thus enabling these two reporter cell lines to be used for screening PDE inhibitors. Figure 8 shows the principles of this new screening method. To improve the detection efficiency, AC/GC agonists were also introduced into this detection system.

Through practical testing, the new method was shown to detect the activity of various types of PDE inhibitors, but the detection window of cGMP was narrower than that of cAMP. The possible reason was believed to be the concentration difference between cAMP and cGMP under physiological conditions. The content of cAMP in tissues is about ten times or even tens of times that of cGMP [31]. A low content of cGMP may indicate a slower production rate and may also result in a smaller range of the dynamic changes that it can cause in the response pathway. In addition, the IC_50_ values of some PDE inhibitors measured in the experiment were higher than the actual values detected in their in vitro enzyme activity experiments. The reason for this phenomenon is considered to be that the activity detection of inhibitors in the new method is carried out in living cells. For the natural product icariin, this may be due to its high molecular weight, poor solubility, and difficulty in penetrating the cell membrane [32]. However, this indicated that the new method could directly screen compounds that can effectively enter the cell membrane. PDE2 is a class of non-selective cyclic nucleotide hydrolases for which PF-999 has a favorable inhibitory effect. This was significantly different from our detection values. According to previous studies, PDE2 is significantly expressed in the brain, heart tissues, and immune cells [26]. HEK293T cells, as human embryonic kidney cells [33], may have very limited PDE2 content, making it difficult to detect the effects of PDE2 activity inhibition. As for the broad-spectrum PDE inhibitor IBMX, the IC_50_ of IBMX detected using both screening models was higher than the actual value. This might be due to the presence of many PDE subtypes in cells that can be inhibited by IBMX, requiring higher concentrations to fully inhibit all subtypes, resulting in a delayed plateau phase.

Overall, a new method for screening PDE inhibitors has been developed and its effectiveness has been successfully validated. The active compounds screened using this method have also been shown to have varying degrees of inhibitory activity against PDE4B and PDE7A. Compared with traditional PDE inhibitor screening methods, this method is cleaner, safer, and more easily operable. Since the materials involved in the experiment are almost non-toxic to cells, experiments can be conducted in live cells. This enables the method to detect dynamic changes in cAMP/cGMP levels in live cells over time under the action of PDE inhibitors, which is more conducive to observing the physiological response of inhibitors. More importantly, the raw materials used in the experiment are relatively inexpensive and easy to obtain. The large capacity of the 96-well plate allows for the simultaneous screening of a large number of compounds, greatly reducing the experimental cost and making this method more suitable for the high-throughput screening of PDE inhibitors. In addition, since cAMP and cGMP cell screening models are used, it is possible to preliminarily determine whether the compound is a cAMP-specific PDE inhibitor, a cGMP-specific PDE inhibitor, or a dual-substrate PDE inhibitor by determining whether the compound shows activity on a single model or both. This guides the next steps of the experiment, narrowing the detection range of PDE subtypes and further saving the cost of subsequent experiments. Of course, as the new method is based on the cellular level, in addition to PDE, other pathways can affect intracellular cAMP/cGMP levels, such as AC/GC. Therefore, this method cannot be used to directly determine whether the ability of the tested compound to increase the intracellular cyclic nucleotide content is due to its inhibition of PDE. However, before being added to the sample, the cells had already been treated with high concentrations of AC/GC agonists, which to some extent reduced the impact of both on the experimental results. In subsequent experiments, the screened compounds will also be measured using PDE Glo technology to ultimately determine their inhibitory activity against different subtypes of PDE.

## 5. Conclusions

In summary, we developed a new method for the screening of PDE inhibitors and successfully verified its effectiveness in practical applications. The active compounds screened through this new method were confirmed to have varying degrees of inhibitory activity against PDE4B and PDE7A. Compared with the traditional PDE inhibitor screening methods, its safety, strong operability, and low costs make it more suitable for the high-throughput screening of PDE inhibitors, with good application prospects and development space.

## Figures and Tables

**Table 1:** The activity of compounds detected using the new method.

Compound	Luminescence Signal (%)
Rolipram	300
**1**	109
**2**	51
**3**	87
**4**	77
**5**	55
**6**	51
**7**	53

**Table 2:** Experimental IC_50_ (μM) of PDE4B and PDE7A.

Compound	PDE4B	PDE7A
Rolipram	0.1	-
BRL-50481	-	0.2
**1**	63.7	28.7
**2**	25.5	47.4
**3**	5.7	4.5
**4**	12.6	8.6
**5**	14.1	2.1
**6**	-	36.2
**7**	28.2	5.6

## Data Availability

Data are contained within the article and Supplementary Materials.

## Supplementary Materials

The following supporting information can be downloaded at: https://www.mdpi.com/article/10.3390/bios14050252/s1. Figure S1: Influence of AC/GC agonist concentration on detection results. (**A**) When different concentrations of the AC agonist forskolin were added, the cell screening model for cAMP showed the detection results of rolipram (5 μM) and PBS. (**B**) When different concentrations of the GC agonist SNAP were added, the cell screening model for cGMP showed the detection results of the PDE5 inhibitor sildenafil (10 μM) and PBS. The data represent the highest luminescence signals detected and are expressed as means ± standard errors (n = 3), **, *p* < 0.01 vs control; ***, *p* < 0.001 vs control.
