# Supplementary material for: The Construction and Application of a New Screening Method for Phosphodiesterase Inhibitors
Source: Biosensors (Basel). 2024 May 16;14(5):252. doi: 10.3390/bios14050252 (PMC11117652; doi:10.3390/bios14050252)
Supplement: Supplementary file 1 [file biosensors-14-00252-s001.zip › biosensors-2968447-supplementary.pdf]

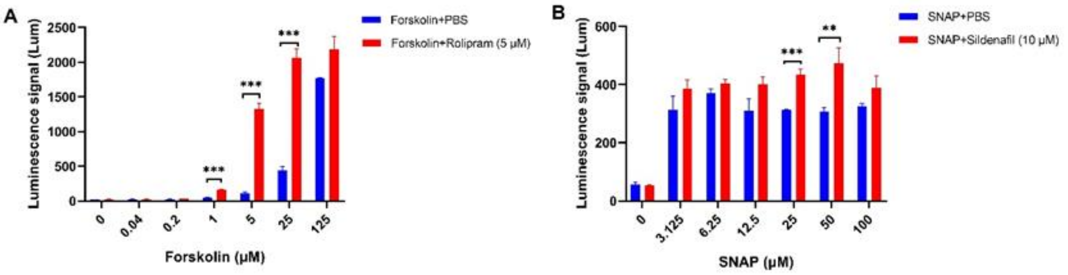

Figure S1. Influence of AC/GC agonist concentration on detection results. (A) When different concentrations of the AC agonist forskolin were added, the cell screening model for cAMP showed the detection results of rolipram (5 μM) and PBS. (B) When different concentrations of the GC agonist SNAP were added, the cell screening model for cGMP showed the detection results of the PDE5 inhibitor sildenafil (10 μM) and PBS. The data represent the highest luminescence signals detected and are expressed as means ± standard errors (n = 3), **, $p < 0.01$ vs control; ***, $p < 0.001$ vs control.
